# Evaluation and Recommendations for Routine Genotyping Using Skim Whole Genome Re-sequencing in Canola

**DOI:** 10.3389/fpls.2018.01809

**Published:** 2018-12-07

**Authors:** M. Michelle Malmberg, Denise M. Barbulescu, Michelle C. Drayton, Maiko Shinozuka, Preeti Thakur, Yvonne O. Ogaji, German C. Spangenberg, Hans D. Daetwyler, Noel O. I. Cogan

**Affiliations:** ^1^Agriculture Victoria, AgriBio, Centre for AgriBioscience, Bundoora, VIC, Australia; ^2^School of Applied Systems Biology, La Trobe University, Bundoora, VIC, Australia; ^3^Agriculture Victoria, Grains Innovation Park, Horsham, VIC, Australia

**Keywords:** GBS, low coverage, *Brassica napus*, doubled haploid, plant

## Abstract

Whole genome sequencing offers genome wide, unbiased markers, and inexpensive library preparation. With the cost of sequencing decreasing rapidly, many plant genomes of modest size are amenable to skim whole genome resequencing (skim WGR). The use of skim WGR in diverse sample sets without the use of imputation was evaluated *in silico* in 149 canola samples representative of global diversity. Fastq files with an average of 10x coverage of the reference genome were used to generate skim samples representing 0.25x, 0.5x, 1x, 2x, 3x, 4x, and 5x sequencing coverage. Applying a pre-defined list of SNPs versus *de novo* SNP discovery was evaluated. As skim WGR is expected to result in some degree of insufficient allele sampling, all skim coverage levels were filtered at a range of minimum read depths from a relaxed minimum read depth of 2 to a stringent read depth of 5, resulting in 28 list-based SNP sets. As a broad recommendation, genotyping pre-defined SNPs between 1x and 2x coverage with relatively stringent depth filtering is appropriate for a diverse sample set of canola due to a balance between marker number, sufficient accuracy, and sequencing cost, but depends on the intended application. This was experimentally examined in two sample sets with different genetic backgrounds: 1x coverage of 1,590 individuals from 84 Australian spring type four-parent crosses aimed at maximizing diversity as well as one commercial F_1_ hybrid, and 2x coverage of 379 doubled haploids (DHs) derived from a subset of the four-parent crosses. To determine optimal coverage in a simpler genetic background, the DH sample sequence coverage was further down sampled *in silico*. The flexible and cost-effective nature of the protocol makes it highly applicable across a range of species and purposes.

## Introduction

Advances in next-generation sequencing have enabled the application of genomics for the improvement of agronomically important crop species. Genomic selection (GS) and genome-wide association studies (GWAS) have delivered considerable crop improvements and rely on high density markers spread throughout the genome (Goddard and Hayes, 2007; Desta and Ortiz, 2014). Genotyping-by-sequencing (GBS) in the form of target capture and complexity reduction methods have greatly facilitated the use of genomics by cost-effectively providing dense SNP markers (Mamanova et al., 2010; Davey et al., 2011; Hirsch et al., 2014). Target capture methods have been mostly applied in the form of SNP chips and although they can be cost-effective in commonly studied species such as cattle, they are often expensive or unavailable in crop species, are unable to identify novel loci, and any genetic inferences made are influenced by the initial SNP discovery method (Thomson, 2014; Rasheed et al., 2017). Conversely, complexity reduction methods are generally cost-effective and easy to implement with few prior genomic resources required (Scheben et al., 2017). The Elshire et al. (2011) method of GBS through restriction-site associated DNA (GBS-RAD) is commonly used in crop species but results in high missing data, often calls variants in a dominant manner due to the presence-absence nature of enzymatic cut sites and struggles to identify heterozygotes (Hirsch et al., 2014). The other complexity reduction method, GBS-transcriptomics (Malmberg et al., 2018a) relies on mRNA, which must be of high quality and only delivers SNPs within the exome (Scheben et al., 2017).

While such methods can be cost-effective, especially in species with large genomes, the modal genome size of plants is approx. 600 Mbp (Gregory, 2005), such that many species with a reference genome can be cost-effectively genotyped by skim whole genome re-sequencing (skim WGR). Although there is currently no convention, we consider skim WGR to be anything less than 10x genome coverage. Skim WGR is a flexible, high-throughput, high-density marker system with unbiased representation of the whole genome, and cheaper sample preparation than other popular GBS methods (Huang X. et al., 2009; Rowan et al., 2015). A substantial advantage of the system is the ability to control marker density by altering sequencing depth, with sequencing costs varying linearly. The balance of coverage versus sample number needs to be considered when choosing an appropriate sequencing depth.

Skim WGR has been primarily adopted in rice since the introduction of the method by Huang X. et al. (2009) for linkage mapping in rice recombinant inbred lines (RILs) sequenced to an ultra-low coverage of 0.02x. Due to the pitfalls of low-coverage sequencing, SNP calling between the deeply sequenced parental genomes and a sliding window method were used to collectively determine the genotypes of the low-coverage sequences. Since then a parent-independent inference method for use in biparental breeding populations has been developed in a rice RIL population (Xie et al., 2010). Numerous other studies have applied skim WGR in biparental plant populations, with various bioinformatics methods and at a range of sequencing coverage (0.055x–4x), including rice (Gao et al., 2013; Ma et al., 2016; Zhou et al., 2016; Jiang et al., 2017; Zhang et al., 2017), sorghum (Zou et al., 2012), foxtail millet (Ni et al., 2017), chickpea (Bayer et al., 2015; Kale et al., 2015), safflower (Bowers et al., 2016), pea (Boutet et al., 2016), soybean (Xu et al., 2013; Karthikeyan et al., 2017; Lu et al., 2017), *Arabidopsis thaliana* (Rowan et al., 2015), melon (Hu et al., 2018), potato (Marand et al., 2017), and a doubled haploid (DH) mapping population of canola (Bayer et al., 2015). Other applications of skim WGR include the improvement of the foxtail millet reference genome (Ni et al., 2017) and the *B. napus* Darmor-*bzh* reference genome (Bayer et al., 2017). It is also possible to call SNPs from low-coverage sequences in diverse sample sets. Huang et al. (2010) performed *de novo* SNP discovery in 517 rice varieties sequenced to approx. 1x coverage. Comparison with 4 deeply sequenced cultivars found genotype call accuracy above 99.9%, with 20.1% of SNPs from the deeply sequenced individual re-called in the same individual sequenced to 1x coverage. Skim WGR of diverse samples has been applied in rice (Huang et al., 2012a,b; Chen et al., 2014, 2018; Wang et al., 2016; Dong et al., 2018), foxtail millet (Jia et al., 2013), maize (Jiao et al., 2012), *B. oleracea* and was evaluated using a computational simulation approach in *B. rapa* (Fu et al., 2016).

As skim WGR results in missing data in the form of absent markers and incomplete allele recovery, the above-mentioned studies have often employed some form of specialized SNP discovery and genotyping method. Many studies have been able to exploit the ability to make assumptions based on biparental population structure, and even in diverse sample sets, efforts have been made to improve SNP genotyping. Having sequenced 533 diverse rice varieties, Chen et al. (2014) used 950 1x coverage sequences from Huang et al. (2012b) to improve SNP genotyping and imputation in their own samples. Fu et al. (2016) used a pooled mapping approach to call SNPs in *B. rapa* and *B. oleracea*, before genotyping these SNPs in individuals. Restricting genotype calling to a list of previously validated SNPs would significantly ease SNP genotyping in low-coverage samples, and simply require the removal of SNPs which are not informative in the sample set of interest, due to low minor allele frequency (MAF) or high missing data. As such the importance of high quality genomic resources shared by crop communities becomes increasingly important and applicable.

Several studies, particularly those applying ultra-low sequencing coverage (<1x) have relied on imputation to achieve sufficient SNPs for linkage mapping and GWAS. The ability to impute from low marker density to high, whether from low to high density array based SNPs or some form of low-coverage GBS, has been widely investigated, particularly in human and livestock studies, which found benefits in sequencing a large number of individuals at low coverage for the application of GS and GWAS (Li et al., 2011; Huang et al., 2012c; Buerkle and Gompert, 2013; Druet et al., 2014; Gorjanc et al., 2015; VanRaden et al., 2015). Skim WGR studies performed in mammals were a 2.9x coverage of bulls for the detection of copy number variation (Keel et al., 2016) and a 0.15x coverage of mice for QTL analysis (Nicod et al., 2016). In contrast to most plant studies, human studies are largely concerned with the ability to detect rare variants causing disease (Li et al., 2011; Pasaniuc et al., 2012; Spiliopoulou et al., 2017), and genotypic imputation in humans and livestock is often bolstered by significant pedigree information. In plants, the accuracy of imputation can vary substantially based on genetic complexity such that imputation in rice, which is almost fully homozygous and has a reference, outperforms imputation in alfalfa, which has heterozygotes and used the genome of closely related species as a reference (Nazzicari et al., 2016). As such, imputation is a valuable and highly accurate tool where the genetics or parentage is known, but is prone to increased errors with novel samples, presenting substantial obstacles for accurate imputation in plant breeding programs. In such cases, the application of skim WGR without the use of additional imputation may be preferable. For applications which require low levels of missing data, it has been shown that 50% missing data in hexaploid wheat leads to sufficiently accurate imputation (Rutkoski et al., 2013). Even when imputed up from up to 80% missing data, GS performed in wheat outperformed phenotypic selection (Rutkoski et al., 2015).

Despite successful application in numerous plant species, skim WGR has not yet been fully exploited in amenable crop species with relatively small genomes as a routine genotyping tool outside of biparental population studies. Cao et al. (2016) sequenced 129 peach accessions to an average coverage of 4.21x, and imputed SNPs for QTL identification. Two studies used 5x coverage of the cotton genome to perform population genetics analyses (Fang et al., 2017a) and GWAS for fiber quality and yield traits (Fang et al., 2017b). Both the peach genome and the cotton genome are smaller than 1 Gbp (Verde et al., 2013; Zhang et al., 2015) and would benefit from the application of skim WGR at lower coverage, sequencing more individuals at a similar cost to increase the power of association studies. Other crop species with genomes smaller than 1 Gbp as well as a reference or draft genome, and so would be ideal candidates for skim WGR include, cassava (Bredeson et al., 2016), cucumber (Huang S. et al., 2009), sugar beet (Dohm et al., 2014), apple (Velasco et al., 2010; Daccord et al., 2017), common bean (Schmutz et al., 2014), flax (Wang et al., 2012), and tomato (The Tomato Genome Consortium, 2012). Even in the absence of a reference genome, deep sequencing of a single individual has been shown to be sufficient to produce a draft genome, and subsequent production of a linkage map in a population of safflower RILs (Bowers et al., 2016). Several plant species which have already applied some form of skim WGR have genomes larger than 1 Gbp (soybean, safflower, pea and maize), such that even plant species with large genomes can apply this technique. As the cost of sequencing continues to decrease, more species will become amenable to skim WGR and existing protocols will become cheaper, allowing for the production of higher quality data at the same cost by increasing sequencing coverage and/or the number of samples.

The aim of the current study was to determine the effect of sequencing coverage, minimum read depth and maximum missing data filtering on SNP genotyping, and apply the method in a range of genetic backgrounds of varying complexity, to determine the applicability of skim WGR as a routine genotyping tool in canola, a highly duplicated allotetraploid with a genome size of 1.13 Gbp, of which 850 Mbp is covered in the Darmor-*bzh* reference genome (Chalhoub et al., 2014). Skim WGR was evaluated in a global diversity panel of 149 canola samples, at seven levels of sequencing coverage (0.25x, 0.5x, 1x–5x), without the use of imputation to exemplify working with novel uncharacterized germplasm and demonstrate in a conservative manner the outputs of the method. As insufficient read sampling is expected to have a significant effect, a range of depth filtering was evaluated (dp 5, dp 4, dp 3 and dp 2), as well as the use of a list of previously validated SNP loci versus *de novo* SNP discovery. Skim WGR was experimentally validated in two populations representing different levels of complexity: first in a highly heterozygous set of 1,590 individuals derived from 84 Australian spring type four-parent crosses aimed at maximizing diversity as well as one commercial F_1_ hybrid, to exemplify the applicability of this method in a complex genetic background. However, as such a diverse set is currently unlikely to be routinely genotyped in canola, 379 DHs derived from 19 of the four parent inter-crosses and which have inter-relationships, were sequenced to 2x coverage and further computationally sub-setted to determine whether a lower level of skim WGR is feasible in a simpler genetic background.

## Materials and Methods

### Global Diversity Panel

#### *In silico* Generation of Fastq Files

The fastq files of 149 canola samples sequenced by Malmberg et al. (2018b): available at NCBI BioProject accession number PRJNA435647) were used to produce skim WGR sequences *in silico*, for analysis of the method. The original fastq files had an average of 10x coverage of the Darmor-*bzh* reference genome (Chalhoub et al., 2014) and regardless of actual coverage, each sample was assumed to have 10x coverage and was computationally sub-setted based on the number of reads to produce skim WGR fastq files representing 0.25x, 0.5x, 1x, 2x, 3x, 4x, and 5x coverage of the reference (Supplementary Figure S1). Using this method, there is a range in coverage between samples such as would be experienced in a real data set, more accurately representing the applicability of skim WGR by incorporating the variability in coverage produced by pooled sequencing.

#### Bioinformatics Analysis

All skim fastq files were quality and adaptor trimmed, then aligned to the *B. napus* Darmor-*bzh* whole genome reference (Chalhoub et al., 2014) using BWA and the MEM algorithm (v0.7.12: Li, 2013) to produce BAM files. Reads were also filtered for a minimum mapping quality of 30 to remove reads aligning to multiple locations in the reference genome. SNPs were called using SAMtools mpileup (v0.1.19-44428cd: Li et al., 2009) with a lenient list of approx. 9.4 million SNPs to capture as much of the variation present as possible (Malmberg et al., 2018b), and converted to a VCF file of biallelic SNPs using BCFtools view (v0.1.19-44428cd: Li et al., 2009) and VCFtools (v0.1.12a: Danecek et al., 2011).

Filtering on the resulting VCF files, in all instances, was performed in R (v3.1.2: R Development Core Team, 2012) after conversion to genotype and depth matrices. To assess the effect of sequencing depth, a range of minimum read depths including a minimum read depth of 5, 4, 3 and 2 were applied to each of the skim levels, resulting in 28 list-based SNP sets (Supplementary Figure S1). All list-based SNP sets were additionally filtered for maximum missing data of 0.5, minimum MAF of 0.05 [calculated separately for spring and remaining diverse global samples as suggested by Malmberg et al. (2018a)] and maximum heterozygosity of 0.1. In all instances, each of the resulting skim SNP sets were compared to the original 10x sequencing data to determine genotype accuracy. If the skim SNP set was filtered to a minimum read depth of 2, the comparison was made against the original 10x sequencing data filtered to a minimum depth of 2, and so on for each skim sequencing coverage level.

To assess the potential for *de novo* SNP discovery, the skim BAM files were also processed for variant discovery with mpileup omitting a SNP list, converted to VCF files, then genotype and depth matrices, and filtered in R using the same parameters as described above, resulting in 28 *de novo* SNP sets. Due to the size of some of the VCF files resulting from *de novo* SNP discovery, additional filtering for a minimum read depth of 2 and maximum missing data of 0.5 was applied using VCFtools prior to converting the VCF files to genotype and depth matrices and performing all further filtering in R.

### Experimental Demonstration of *in silico* Predicted Optimal Skim WGR

#### Four Parent Crosses

An experimental validation was performed using 1,590 individuals, derived from 84 different four-parents crosses, that used 97 Australian spring type canola varieties as well as one commercial F_1_ hybrid, as the parent material. Libraries were prepared using the method described by Malmberg et al. (2018b) for WGR libraries. Samples were pooled and run on an Illumina Hiseq 3000 to generate approx. 1 Gbp of sequencing data per sample.

BAM files were generated in the same way as described above. SNP genotyping was performed by supplying the SAMtools mpileup algorithm with a list of over four million high-confidence SNPs, previously validated in Malmberg et al. (2018b), before filtering for minimum read depths ranging from 2 to 5, MAF of 0.01 and maximum missing data of 0.5.

#### Doubled Haploids

A second experimental evaluation was performed in 379 DH samples derived from 19 of the four-parent crosses. Whole genome libraries were prepared using the method for WGR libraries described by Malmberg et al. (2018b), modified by substituting the REPLI-g mini kit for the UltraFast REPLI-g Mini Kit (QIAGEN) and using the JetSeq Flex DNA Library Preparation Kit (Bioline) from the dA-tail step through to the final PCR amplification. Samples were sequenced on an Illumina Hiseq 3000 aiming to generate approx. 2.5 Gbp of raw sequencing data per sample.

A range of skim coverage levels were evaluated in the DH data set to determine the impact of sequencing coverage on the number and accuracy of captured genotypes in a simpler genetic background. The same method as described above for the global diversity panel was applied to generate 0.25x, 0.5x, and 1x skim coverage fastq files for each of the DH samples. BAM files were generated in the same way as described above. SNP genotyping was performed by supplying the SAMtools mpileup algorithm with the list of over four million high-confidence SNPs, removing triallelic SNPs but retaining all other SNPs, regardless of whether they were variant in the population. Finally, each skim coverage level was filtered for minimum read depths of 1, 2, 3, 4, and 5, resulting in 15 skim DH genotype matrixes (0.25x dp 1 to 5, 0.5x dp 1 to 5, 1x dp 1 to 5). Each of the resulting skim DH genotype matrixes were compared to the original 2x DH sequencing data to determine genotype accuracy. If the skim DH set was filtered to a minimum read depth of 1 or 2, the comparison was made against the original 2x sequencing data filtered to a minimum depth of 2, and if the skim DH set was filtered to a minimum read depth of 3, the comparison was made against the original 2x sequencing data filtered to a minimum depth of 3, and so on for each filtering depth.

## Results

### *In silico* Evaluation of Skim WGR in Diverse Canola Varieties

#### Average Genome Coverage and List-Based High-Confidence SNP Markers

**FIGURE 1:**
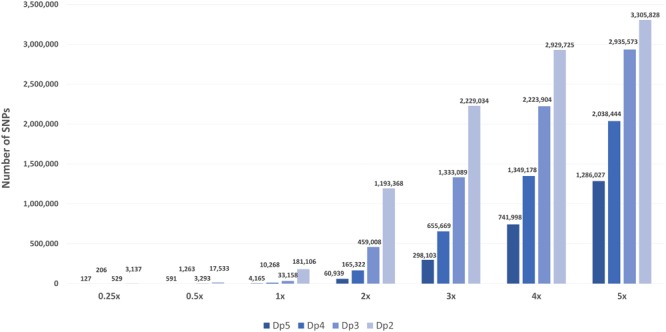
The number of high-confidence SNPs in the global diversity panel for each skim coverage level (0.25x, 0.5x, 1x–5x) and minimum read depth (dp 5, dp 4, dp 3, and dp 2).

The BAM files generated from the original 10x sequences had an actual average genome coverage of 9.27x. The average reference genome coverage of the *in silico* skim BAM files aiming for 0.25x, 0.5x, 1x, 2x, 3x, 4x, and 5x were 0.19x, 0.38x, 0.77x, 1.54x, 2.31x, 3.08x, and 3.85x, respectively, with a varying range of depth around the mean (coefficient of variation of 0.27 for all skim coverages), more representative of an actual pooled sequenced data set. Increasing sequence coverage resulted in an exponential increase (*R*^2^ = 0.9289–0.9998) in SNPs and relaxing the stringency of depth filtering also significantly increased the total number of SNPs for each of the skim levels (Figure 1).

**FIGURE 2:**
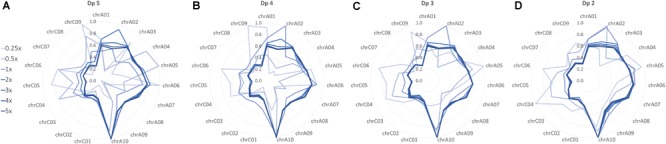
Relative distribution of high-confidence SNPs across chromosomes in the global diversity panel skim SNP sets filtered to a minimum read depth of **(A)** dp 5, **(B)** dp 4, **(C)** dp 3, and **(D)** dp 2. SNP density between chromosomes within each SNP set (skim coverage level and depth filter combination) was compared, with the highest SNP density assigned a value of 1 and all other chromosomes assigned a value relative to this.

Relative SNP density was compared between chromosomes to determine the effect of skim coverage and filtering stringency on marker spread. For the ultra-low skim coverage levels (0.25x and 0.5x), stringent depth filtering resulted in highly variable SNP density, with some chromosomes having one or no SNPs (Figure 2A and Supplementary Figure S2). Relaxing depth filtering improved marker spread, resulting in more even coverage of chromosomes (Figures 2B–D and Supplementary Figure S2). Relative SNP density was similar between the higher skim coverage levels (1x–5x), with a higher SNP density found on the A genome chromosomes, and marginal improvement in marker spread for relaxed depth filtering (Figures 2A–D and Supplementary Figure S2). This suggests that beyond 1x sequencing coverage, SNP markers are being evenly sampled across the genome. Sampled SNP distribution largely matches the distribution of the nine million markers in the SNP list as confirmed by heatmap plots of SNP density in 1 Mbp bins (Supplementary Figure S2).

#### Accuracy of Genotype Calls Compared to Original 10x Data Set

As low sequencing depth is expected to impact the accuracy of genotype calls, a comparison was made between the original 10x data set and the skim data in each of the 28 list-based high-confidence SNP sets: 0.25x, 0.5x, 1x–5x, each filtered to a depth of 2, 3, 4, and 5. This relies on the assumption that the genotypes are correct in the full 10x data.

**FIGURE 3:**
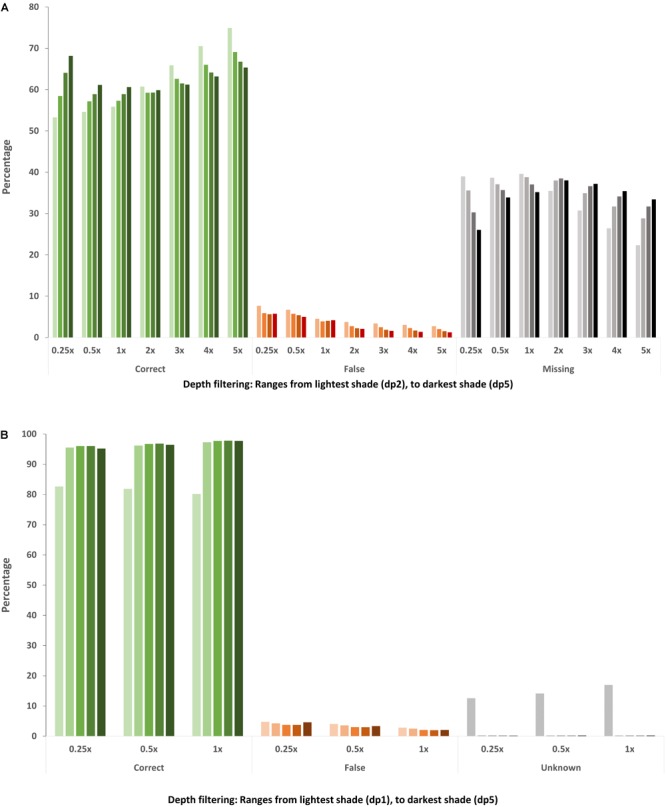
Accuracy of genotype calls in the **(A)** *in silico* skim global diversity panel compared to the original 10x sequencing data and **(B)** DH skim data sets compared to the original 2x DH sequencing data. The percentage of genotypes across the whole genotype matrix (each SNP in each individual) within each skim SNP set is represented. Green bars represent genotype calls which are consistent with the corresponding full sequencing data, orange bars represent genotype calls which do not match, and the gray/black bars indicate missing genotypes in the **(A)** *in silico* skim global diversity panel and **(B)** genotypes present in the DH skim data but which could not be evaluated for accuracy due to insufficient read depth in the original 2x data.

In all list-based SNP sets the majority of genotype calls were correct, with matching genotype calls ranging from 53.3–74.9%, while the number of false genotype calls was low (<8%), decreasing as sequencing coverage increases (Figure 3A and Supplementary Table S1). Most of the differentiation from the 10x genotype calls was due to an increase in missing data, ranging from 22.3–39.6%. Disregarding missing data points, the proportion of correct genotype calls ranged from 87.4–98.1% with a corresponding range of false genotype calls from 1.9–12.6%. There are three factors to consider when determining overall genotyping accuracy: correct, missing and false genotype calls. Applying a more stringent filtering depth results in significantly fewer SNPs but for those SNPs that are called, there was generally increased overall accuracy with fewer false calls, less missing data and more correct calls, with the exception of sequencing coverage 2x and above. While increasing the stringency of depth filtering still resulted in fewer false genotype calls at 2x and above, a large proportion of accurate genotypes are also removed, with a corresponding increase in missing data (Figure 3A and Supplementary Table S1). This is likely because SNP calling software uses all available reads to call genotypes and actual sequencing coverage of skim sets of 2x and above is greater than the minimum 2 read cut off, unlike 1x and below data sets where filtering depth is greater than or equal to expected coverage.

As the acceptable amount of missing data within a genotype matrix will vary between studies depending on the intended application, the number of SNPs and corresponding accuracy of genotype calls has been examined in cumulative increments of 10% missing data (Table 1). In this way, an appropriate level of sequencing coverage and minimum depth filtering requirement can be chosen by balancing the total number of informative markers and potential associated loss in accuracy against sequencing requirements. Total marker number behaves as expected, increasing as sequencing coverage and maximum missing data increases, and as filtering depth is relaxed. The variation in overall accuracy within a sequencing coverage level (e.g., 1x) is minimal when coverage ≥1x (1.4–2.4%) but is higher at ultra-low coverages (4.2–5.8%). At ultra-low sequencing coverages, overall accuracy is increased both as filtering depth stringency is increased and less missing data is accepted. Although this strategy will improve accuracy, it results in a significant decrease in SNP markers. At 1x coverage, accuracy is highest when up to 50% missing data is accepted, by a small margin across all filtering depths. At sequencing coverage of 2x and above, accuracy is highest at dp 5 and 50% missing data but is lowest at dp 2 and 50% missing data, suggesting that as depth filtering is relaxed at sequencing coverage >2x, it is beneficial to select markers with less missing data. This strategy is likely favorable as relaxing depth filtering also substantially increased the number of SNP markers available.

**Table 1:** Cumulative number of SNPs (% accuracy of called genotypes) in 10% increments of maximum missing data accepted per SNP, for each sequencing coverage level and minimum depth filter, in the skim global diversity panel.

		Maximum missing data				
Skim level	Minimum read depth	<10%	<20%	<30%	<40%	<50%
0.25x	5	39 (92.1)	54 (91.9)	65 (92.1)	84 (91.9)	127 (92.2)
	4	42 (93.2)	63 (92.5)	76 (92.5)	129 (92.2)	206 (91.9)
	3	50 (92.9)	79 (91.9)	132 (91.4)	264 (91.1)	529 (90.8)
	2	70 (91.6)	180 (90.6)	496 (89.4)	1,326 (88.3)	3,137 (87.4)
0.5x	5	62 (93.2)	104 (92.9)	188 (92.9)	343 (92.6)	591 (92.5)
	4	79 (92.5)	165 (92.4)	346 (92.2)	655 (91.8)	1,263 (91.6)
	3	128 (92.7)	327 (92.3)	749 (91.6)	1,629 (91.1)	3,293 (90.9)
	2	288 (91.8)	1,161 (90.3)	3,217 (89.5)	7,655 (89.0)	17,533 (89.0)
1x	5	229 (92.8)	559 (93.0)	1,180 (93.2)	2,308 (93.3)	4,165 (93.5)
	4	347 (92.9)	1,039 (93.0)	2,415 (93.1)	4,992 (93.2)	10,268 (93.6)
	3	617 (92.9)	2,375 (92.9)	5,834 (93.1)	14,012 (93.3)	33,158 (93.6)
	2	1,968 (92.4)	8,868 (92.3)	26,345 (92.5)	73,089 (92.5)	181,106 (92.5)
2x	5	1,511 (94.5)	4,900 (95.2)	11,761 (95.8)	27,759 (96.3)	60,939 (96.6)
	4	2,834 (94.8)	10,654 (95.5)	29,677 (96.1)	74,253 (96.3)	165,322 (96.4)
	3	6,193 (95.2)	29,664 (95.8)	88,594 (95.8)	219,662 (95.7)	459,008 (95.6)
	2	22,865 (95.3)	121,324 (95.0)	332,147 (94.7)	697,586 (94.4)	1,193,368 (94.2)
3x	5	6,191 (96.0)	24,883 (96.9)	65,784 (97.3)	149,552 (97.4)	298,103 (97.5)
	4	12,760 (96.5)	58,138 (97.0)	157,952 (97.1)	348,461 (97.1)	655,669 (97.0)
	3	32,584 (96.7)	155,568 (96.7)	399,127 (96.5)	796,368 (96.3)	1,333,089 (96.2)
	2	117,715 (96.3)	480,069 (95.8)	996,611 (95.4)	1,616,262 (95.2)	2,229,034 (95.1)
4x	5	19,832 (97.2)	83,871 (97.7)	209,245 (97.8)	423,721 (97.8)	741,998 (97.8)
	4	42,843 (97.5)	184,749 (97.6)	439,851 (97.6)	832,678 (97.5)	1,349,178 (97.4)
	3	106,892 (97.4)	427,994 (97.1)	914,746 (96.9)	1,539,505 (96.8)	2,223,904 (96.7)
	2	323,132 (96.8)	1,004,739 (96.3)	1,719,151 (96.1)	2,397,603 (95.9)	2,929,725 (95.8)
5x	5	51,017 (97.9)	200,955 (98.1)	451,492 (98.1)	817,461 (98.1)	1,286,027 (98.1)
	4	106,435 (97.9)	401,047 (97.9)	836,977 (97.9)	1,400,574 (97.8)	2,038,444 (97.8)
	3	243,287 (97.7)	805,789 (97.5)	1,488,739 (97.3)	2,227,176 (97.2)	2,935,573 (97.1)
	2	618,121 (97.2)	1,545,888 (96.8)	2,329,718 (96.6)	2,959,233 (96.5)	3,305,828 (96.5)

#### *De novo* SNP Discovery

All skim BAM files also underwent *de novo* SNP discovery and filtering, resulting in 28 *de novo* SNP sets. In all instances, *de novo* SNP discovery increased the total number of SNPs (Table 2) compared to the corresponding list-based SNP set. The most significant increase in the total number of SNP markers was observed at ultra-low coverages and relatively stringent depth filtering, with a 44-fold, 37-fold, 19-fold, and 19-fold increase (0.25x dp 5, 0.25x dp 4, 0.25x dp 3, and 0.5x dp 5, respectively), and all other *de novo* SNP sets generated between 13,396 and 344,248 additional SNPs. In all instances, the majority of loci retained in the list-based SNP sets were re-identified, with more than 97% of list-based SNPs identified in the *de novo* discovery exercise (Table 2).

**Table 2:** Number of filtered SNPs generated through *de novo* SNP discovery in the skim global diversity panel.

Skim level	Minimum read depth	No. of SNPs in *de novo* skim sets	% of list-based SNPs identified by *de novo* discovery	Fold increase in SNP no.	Increase in SNP no.
0.25x	5	5,591	100	44.0	5,464
	4	7,573	100	36.8	7,367
	3	10,310	100	19.5	9,781
	2	16,674	99.94	5.3	13,537
0.5x	5	11,224	100	19.0	10,633
	4	14,659	99.92	11.6	13,396
	3	20,245	99.97	6.1	16,952
	2	39,520	99.99	2.3	21,987
1x	5	23,157	99.95	5.6	18,992
	4	33,985	99.98	3.3	23,717
	3	63,924	99.99	1.9	30,766
	2	225,924	99.998	1.2	44,818
2x	5	100,028	99.98	1.6	39,089
	4	220,638	99.99	1.3	55,316
	3	541,071	99.997	1.2	82,063
	2	1,309,224	99.999	1.1	115,856
3x	5	354,623	97.28	1.2	56,520
	4	732,973	97.72	1.1	77,304
	3	1,429,815	97.81	1.1	96,726
	2	2,354,165	98.34	1.1	125,131
4x	5	825,813	97.59	1.1	83,815
	4	1,456,258	97.83	1.1	107,080
	3	2,348,359	97.92	1.1	124,455
	2	3,121,869	98.50	1.1	192,144
5x	5	1,394,073	97.65	1.1	108,046
	4	2,167,536	97.80	1.1	129,092
	3	3,080,766	97.9	1.0	145,193
	2	3,650,078	98.59	1.1	344,248

The genotypes of SNPs identified in the 1x and 5x *de novo* exercise, but which were absent from the corresponding list-based SNP set, were examined in the original 10x data to determine why they did not pass quality filtering in the original 10x sequences. A comparison of genotype calls found the majority to be consistent (64.5–69.9%), a moderate percentage were missing (26.3–32.9%) and a small proportion were incorrect genotype calls (2.6–5.8%) in the *de novo* skim sets. An overall increase in mean missing data and some degree of insufficient allele sampling caused by skim sequencing coverage seems to account for the inclusion of novel SNP loci in the 1x and 5x skim *de novo* SNP sets, with the vast majority removed from the original 10x data set during MAF filtering.

### Experimental Demonstration of *in silico* Predicted Optimal Skim WGR

#### Four Parent Crosses

Actual average genome coverage was 0.93x with a coefficient of variation of 0.34. Due to the heterozygous nature of these samples, the more lenient SNP list (approx. 9.4M SNPs) applied in the global diversity panel could not be used, as resulting SNPs need to be further filtered on excess heterozygosity caused by misalignment. Instead, a list of over four million high-confidence SNPs which have been previously validated and filtered on excess heterozygosity was used. As the data set is large (1,590 samples), the relatively high proportion of absent genotypes resulted in the need to remove a large proportion of SNPs due to excess missing data. A range of filtering depths was considered to balance the total number of markers and the ability to accurately call heterozygous loci. A minimum of 5 reads resulted in only 8,538 SNPs, too few for association studies, while a minimum of 4 reads resulted in 19,073 SNPs (Table 3), an acceptable number of markers while still being stringent enough to prevent insufficient allele sampling. Overall, increasing depth filtering stringency results in a smaller percentage of residual missing data within the data set as a whole, but also reduces the total number of data points.

**Table 3:** SNPs remaining from the 4M SNP list, in the four parent crosses after filtering for MAF of 0.01 and maximum missing data of 0.5 for each minimum read depth of 2, 3, 4, and 5.

Minimum read depth	No. of SNPs	% residual missing data	Total data points (SNPs^∗^ individuals)	No. of missing genotypes
5	8,538	33.9	13,575,420	4,607,124
4	19,073	36.5	30,326,070	11,060,070
3	43,799	38.6	69,640,410	26,852,967
2	264,952	39.9	421,273,680	168,083,777

#### Doubled Haploids

Average genome coverage of the original DH sequencing data was 2.1x, with a resulting average coverage of 0.22x, 0.45x, and 0.89x for the 0.25x, 0.5x, and 1x *in silico* skim data sets, respectively, with a coefficient of variation of 0.17 in all skim sets.

This DH set was derived from 19 of the 1,590 four-parent cross samples genotyped and described earlier, with between 1 and 83 DHs generated from each of the 19 source plants. As the intention of this DH set was not to determine the number of informative SNPs, but rather to examine overall accuracy of skim genotype calls in a simpler and more breeding relevant genetic background, all genotype calls from the list of over four million high-confidence SNPs were retained unless the SNP was triallelic, in which case it was changed to missing.

As expected, the average number of genotypes captured in an individual increased with sequencing depth and as depth filtering was relaxed (Table 4), such that in any genetic background, increasing sequencing depth and/or relaxing depth filtering will result in an increase in captured genotypes. Of the genotype positions which had sequencing data available, the majority (dp 1: 80.2–82.7% and dp 2 or greater: 95.2–97.7%) were found to match the corresponding genotype call in the full 2x DH sequencing data, with a small proportion of incongruous calls (2.1–4.8%), even in the skim data sets only requiring a single sequencing read (Figure 3B). However, a number of genotypes in the skim sets, particularly for minimum read depth of 1, could not be compared to the full 2x sequences due to insufficient read depth such that their accuracy is unknown. Furthermore, heterozygous calls were considered to be correct if they matched the corresponding genotype call in the 2x DH sequences, however, in a DH background, heterozygous genotype calls are indicative of error such as read misalignment. The total percentage of called genotypes which were heterozygous was low across all DH skim data sets, but were present and ranged from 3.1 to 6.8%.

**Table 4:** The average, minimum, and maximum number of SNPs genotyped in individuals in the DH samples for each sequencing coverage level and filtering depth.

	No. of SNPs genotyped in individuals			
Skim level	Minimum read depth	Average	Minimum	Maximum
0.25x	5	24,144	666	47,800
	4	56,014	1,531	104,674
	3	82,229	2,030	154,165
	2	243,102	13,850	404,436
	1	426,370	31,188	700,290
0.5x	5	62,917	1,216	128,273
	4	127,748	2,957	241,270
	3	189,495	4,062	360,101
	2	460,405	26,899	740,728
	1	755,794	60,435	1,192,737
1x	5	173,476	2,294	358,718
	4	299,618	5,920	553,618
	3	429,318	8,738	787,243
	2	836,761	53,316	1,292,317
	1	1,254,796	117,965	1,860,615
2x	5	511,008	6,083	983,954
	4	736,420	14,846	1,281,324
	3	969,265	23,991	1,611,694
	2	1,489,570	118,138	2,144,288

## Discussion

The present study has found skim WGR to be applicable in canola, an allotetraploid with a relatively modest genome size, in a range of genetic backgrounds. The application of skim WGR is significantly eased in canola due to the availability of a reference genome, covering c. 850 Mbp of the 1.13 Gbp genome (Chalhoub et al., 2014), and a list of previously validated SNPs (Malmberg et al., 2018b). It should be noted that the SNP list used here was developed in 149 whole genome sequences (Malmberg et al., 2018b), which were also used for the *in silico* creation of the global diversity panel skim fastq files used in this study. The application of a SNP list developed outside the sample set of interest may reduce the total number of informative SNPs, highlighting the benefit of further developing SNP resources for canola. Throughout, this study has referred to the expected rather than realized genome coverage based on the proportion of 10x whole genome sequences used to create the skim files. However, in all skim sets, this fell below expectation such that a minimum realized coverage of 1x (between the 1x and 2x sets described here which had a realized coverage of 0.77x and 1.54x, respectively), is likely to be appropriate for most genomics-based studies in canola. Additionally, in an attempt to mitigate the effect of insufficient allele sampling, a range of minimum read depths were used in this study. Often this resulted in selecting SNPs with higher than expected coverage, particularly for ultra-low skim sets. Sequencing depth varies across the genome even with only uniquely aligned reads and has been attributed in part to reference collapse, presence/absence between individuals and minor GC bias but the primary cause of this variance has not been established (Beissinger et al., 2013). As such, the range of minimum read depth examined in this study may result in the selection of some proportion of genetic artifacts being incorporated at low sequencing coverage, but as these markers were genotyped based on a SNP list developed in the set of 10x sequences filtered using an appropriate minimum read depth of 5, this is likely to represent a small portion of SNPs. Furthermore, this is an issue which is likely to affect many GBS systems in a similar way.

As expected, the number of list-based SNPs retained in the global diversity panel skim sets increased exponentially with sequencing coverage and substantially as minimum read depth filtering was relaxed. Similar results have been found in other studies (Nazzicari et al., 2016; Wickland et al., 2017; Abed et al., 2018). The required number of markers will vary significantly between studies and intended application. For example, association studies require the genome to be sufficiently saturated with markers to ensure markers and causal variants are in linkage disequilibrium (LD), which varies significantly between species and populations. As such, LD information of populations can be used to inform on expected required marker density if known. Three list-based SNP sets, 0.5x dp 2, 1x dp 3, and 2x dp 5 resulted in 17K, 33K, and 60K SNP markers, respectively, comparable to or greater than the number of markers typically captured in canola using the *Brassica* 60K array (Li et al., 2014; Qian et al., 2014; Wang et al., 2014; Fomeju et al., 2015; Hatzig et al., 2015) or GBS-RAD methods (Chen et al., 2013; Wu et al., 2016). Although all three sets could be deemed suitable in terms of marker number, three factors should be considered when selecting an optimal skim WGR strategy: the acceptable level of missing data, the effect of genotype error on downstream applications and cost efficiency.

The presence of missing data in skim WGR in the form of absent genotypes often results in the need to perform imputation prior to further downstream analysis. The ability to impute missing data has been well characterized across a range of germplasm types, with high accuracy relatively easily achieved in simple genomes such as rice and biparental populations where parentage is known (Huang et al., 2010, 2012a; Yu et al., 2011; Gao et al., 2013; Rowan et al., 2015; Ma et al., 2016; Wang et al., 2016) and is generally more difficult in complex genomes and for rare variants (Nazzicari et al., 2016). Sufficiently high accuracy has been achieved in the presence of up to 50% missing data in unordered markers in hexaploid wheat, with ordered markers expected to achieve higher accuracy (Rutkoski et al., 2013). Although not significantly affected by missing rate of up to about 60%, imputation accuracy typically improves as missing data decreases (Rutkoski et al., 2013; Bajgain et al., 2016; Nazzicari et al., 2016; Elbasyoni et al., 2018), but benefits from a large number of data points such that imputation in a data set with more markers and higher missing data is preferable to fewer markers with less missing data (Torkamaneh and Belzile, 2015). Furthermore, if the genome is under-saturated the inclusion of markers imputed from high missing data (80%) is beneficial for association studies due to the increase in marker density, despite any potential decrease in accuracy (Rutkoski et al., 2013; Jarquin et al., 2014; Elbasyoni et al., 2018). As low coverage sequencing methods such as GBS-RAD gain popularity due to their cost-efficiency, more focus is being given to imputation in low coverage samples, particularly in plant species (Xie et al., 2010; Swarts et al., 2014; Fragoso et al., 2016; Zheng et al., 2018), which often lack the well-developed resources available in humans and livestock. However, should it be necessary to avoid high missing data, the total number of SNPs and accuracy of genotypes captured has been provided cumulatively in 10% increments to aid with selecting the best balance between acceptable missing data, total number of markers and resulting accuracy for each sequencing coverage level and minimum read depth requirement (Table 1).

As well as absent genotypes, low coverage sequencing is expected to result in some degree of insufficient allele sampling, causing heterozygous genotypes to appear homozygous, such that highly heterozygous populations will be more affected. In addition, sequencing error and other noise which would typically be drowned out by deeper sequencing depth may become incorporated into the genotype matrix. In all list-based global diversity panel skim sets, most genotype calls were correct, with increased missing data and a relatively small percentage (1.2–7.7%) of erroneous genotype calls, which decreased with sequencing coverage and more stringent depth filtering. Although genotype errors were low, the total number of data points varied significantly between SNP sets. For example, relaxing depth filtering from 5 reads to 2 reads in the 2x data increases the total number of data points generated (individuals^∗^SNPs) from 9 million to over 177 million. While the percentage of false genotype calls only increases from 2.1 to 3.7%, the total number of erroneous data points increases from 190K to 6.5 million (Supplementary Table S1). Additionally, more erroneous genotypes may cause false SNPs to be included if *de novo* SNP discovery is used, which will be necessary in species without previously validated high-confidence SNP positions available. As the deeply sequenced genomes of the global diversity panel were available, it was possible to characterize the errors associated with *de novo* SNP discovery in skim WGR. Most legitimate SNPs present in the population should already be incorporated in the SNP list, particularly as a lenient list of over nine million SNPs (Malmberg et al., 2018b) was used. In all instances, *de novo* SNP discovery increased the number of markers, particularly for ultra-low coverage sequencing, while still identifying the majority of SNPs retained in the corresponding list-based SNP set and so is capable of capturing high-confidence SNPs. An overall increase in missing data and false genotype calls in the skim sequences were found to account for new SNPs identified during *de novo* SNP discovery, having been removed from the original 10x data during filtering, but will have less impact on association studies due to a lower average MAF compared to high-confidence list-based SNPs. Of course, this analysis is based on a single SNP discovery software and using different software has been found to affect total number of SNPs discovered, missing data, genotype accuracy and proportion of heterozygotes, with relatively few SNPs common between software (Yu and Sun, 2013; Clevenger et al., 2015; Torkamaneh et al., 2016; Wickland et al., 2017). Studies employing skim WGR and *de novo* SNP discovery should be aware of the potential impact of an increase in erroneous SNPs.

An increase in error has been found to reduce the accuracy and filling rate of imputation (Huang et al., 2010), while the effect of increased error, whether from insufficient allele sampling or imputation errors, on downstream applications needs to be considered. For genetic mapping, the incorporation of false genotype calls has been found to artificially inflate genome size (Bajgain et al., 2016). For GWAS, a small degree of error is likely to be preferable to inadequate marker density as incorporating more markers despite higher error rates results in greater association power (Torkamaneh and Belzile, 2015; Wang et al., 2016), though studies using this approach should be mindful of inflated association. Very low coverage levels have poor association power to detect rare variants and results in more false-positive associations in GWAS (Bajgain et al., 2016; Xu et al., 2017), which could be an effect of errors or inadequate marker density. Nonetheless, at genotype accuracies >98% in rice, it was found that 1x coverage matched 20x in terms of mapping power and ability to detect causal variants (Wang et al., 2014). Genomic selection is more robust to variations in overall error rate, with the choice of imputation method having little effect despite differences in accuracy (Poland et al., 2012; Rutkoski et al., 2013; Jarquin et al., 2014; Elbasyoni et al., 2018), and simulations have shown that unless error is substantial (>10%), GS accuracy is not substantially affected (Perez-Enciso et al., 2015). The lack of effect on GS accuracy may be because most imputation errors affect low frequency variants, although this means the ability to detect rare variants will be diminished (Perez-Enciso et al., 2015). Inability to capture heterozygous genotypes is potentially more problematic as it is essential to capture all variation present for accurate GS (Ashraf et al., 2014, 2016; Gorjanc et al., 2017). Simulations found insufficient allele sampling associated with low coverage sequencing was detrimental to GS at very low coverage levels, but GS became increasingly robust to these errors as marker density increased (Gorjanc et al., 2015), and if higher marker density is achieved through deeper sequencing coverage, it can be expected that any associated insufficient allele sampling will diminish as well. Although increasing genotype accuracy does benefit GS accuracy (Gorjanc et al., 2015), ultimately, sample size both in terms of total markers and individuals sequenced will have a bigger effect on GS performance than the incorporation of a small proportion of erroneous genotypes, as numerous studies have found benefit in sequencing more individuals at lower coverage and quality (Li et al., 2011; Pasaniuc et al., 2012; Buerkle and Gompert, 2013; Ashraf et al., 2014; Druet et al., 2014; Gorjanc et al., 2015; Xu et al., 2017). The majority of studies agree that ultra-low sequencing is often problematic, likely due to high error coupled with low marker density (Gorjanc et al., 2015; Ashraf et al., 2016; Xu et al., 2017; Abed et al., 2018), such that ultra-low sequencing may only ever be appropriate in biparental populations or individuals inbred to the degree that they behave as haploids (Wang et al., 2016), and employing specialized SNP genotyping techniques (Huang X. et al., 2009; Huang et al., 2010; Xie et al., 2010).

Along with the ability to avoid the ascertainment bias present in many SNP chips and the potential to capture causal variants (Meuwissen and Goddard, 2010; Poland et al., 2012; Druet et al., 2014; Bajgain et al., 2016; Elbasyoni et al., 2018), a skim WGR approach is highly cost efficient and sequencing resources can be allocated in a flexible manner. Skim WGR library preparation is among the most cost-effective for GBS systems (Huang X. et al., 2009; Rowan et al., 2015) and is a fixed cost per sample, while sequencing costs increase linearly with sequencing coverage such that under current cost structures, assuming $12 per sample library preparation and $30 per Gb of sequencing data on an Illumina Hiseq system, 100 individuals sequenced to 1x coverage will cost $4,200 and 5x will cost $16,200. As the cost of sequencing diminishes, skim WGR as well as other GBS methods, will become increasingly cost efficient at higher sequencing coverages. Conversely, at a fixed available cost of $50,000, 1,190 individuals can be sequenced to 1x or 309 individuals can be sequenced to 5x. If deeper sequencing efforts can be invested, it is better to deeply sequence the training population and sequence more individuals in the testing population at lower coverage (Weigel et al., 2010; Pasaniuc et al., 2012; Gorjanc et al., 2015; Xu et al., 2017). Additionally, when working with biparental populations, deeply sequencing the parental lines, if available, is highly beneficial, allowing for ultra-low coverage of the offspring (Huang X. et al., 2009; Gao et al., 2013; Bayer et al., 2015; Boutet et al., 2016).

Ultimately the selected skim sequencing approach needs to be tailored to the population of interest. For this reason, the 1,590 highly diverse and heterozygous four parent crosses examined in this study were sequenced to an average of 1x coverage primarily due to cost efficiency, avoiding ultra-low sequencing due to issues with low expected marker density and high error rate. As high heterozygosity rates were expected within this population, filtering on excess heterozygosity as typically performed in canola was avoided, and instead a restricted SNP list already filtered on heterozygosity was used, as well as a relatively stringent minimum read depth (dp 4) in attempt to prevent insufficient allele sampling, while balancing the total number of informative markers (19,073). For association studies in heterozygous samples, avoiding insufficient allele sampling and providing sufficient markers is more important than the proportion of missing data (Ashraf et al., 2014, 2016). Canola varieties and breeding lines are typically highly homozygous due to the use of DHs in breeding programs and may be able to use relaxed depth filtering without significantly impacting the accuracy of genotype calls.

The use of skim WGR in DHs was expected to result in a significant improvement in overall genotype accuracy, due to the simpler genetic background. With the exception of the dp 1 skim sets, the majority of called genotypes were accurate (95.2–97.7%) across DH SNP sets, an improvement at ultra-low coverage compared to the global diversity panel skim SNP sets when missing data is ignored (87.4–98.1%). There were fewer false calls in the DHs (2.1–4.6%) compared to the global diversity panel skims (1.9–12.6%), suggesting an improvement of accuracy in a DH background at some coverage levels, and that ultra-low skim WGR may be feasible in DHs. However, a significant number of loci were heterozygous (3.8–5.1%), decreasing as more stringent depth filtering was applied but not significantly affected by sequencing coverage. This suggests consistent misalignment of short reads, which has been found to occur at a local level in canola (Malmberg et al., 2018a), and emphasizes the need to eliminate these false-positive SNPs by using methods such as filtering on mapping quality and excessive heterozygosity where possible. Ultimately, long read technology is expected to improve alignment, extending far enough to uniquely align to the genome, and as the sequencing accuracy of long read technology improves, may become the optimal GBS method in highly duplicated genomes.

Alternatively, genotype accuracy can be improved in skim WGR through the application of more advanced bioinformatics techniques. A GBS-RAD study in barley using a range of coverage between 16 and 4 average reads per SNP and minimum read depth of 2, achieved genotype accuracies of 99, 97, and 95% at maximum missing data of 15, 50, and 80%, respectively (Abed et al., 2018), similar to the current study where an increase of missing data from 10 to 50% resulted in a 1 and 0.7% drop in accuracy at 4x dp 2 and 5x dp 2, respectively. GBS-RAD studies in soybean, which has a similar genome to canola in terms of size, duplication and expected heterozygosity, have achieved high genotyping accuracy at >98%, with little variation around maximum missing data (Torkamaneh and Belzile, 2015) and, between 95.55% and >99% primarily depending on the GBS pipeline implemented (Wickland et al., 2017). It seems likely that the implementation of the Fast-GBS pipeline (Torkamaneh et al., 2017) employed by each of the abovementioned studies is responsible for the high genotype accuracies achieved, as Fast-GBS involves haplotype construction to improve variant calling. In addition, methods such as the sliding window and inference approach commonly employed in rice studies (Huang X. et al., 2009; Xie et al., 2010), the selection of representative SNPs based on LD and naïve recalling of genotypes in DHs based on allelic proportions, are likely to be of great benefit to skim WGR studies. Although this manuscript aims to establish the effect of sequencing coverage and depth filtering on genotype quality in the absence of such measures, the implementation of appropriate genotype calling methods is advised. For this reason, the computational and bioinformatics requirements associated with skim WGR is greater than SNP chips, but is still likely to be preferable in many species due to the absence of commercially available SNP arrays, significant cost savings, potential for greater marker density, increased association power from capturing causal variants and avoiding SNP array bias.

## Conclusion

This study has demonstrated the applicability of skim WGR outside of biparental segregating populations, in a highly duplicated genome of 1.13 Gbp in size, across a range of genetic backgrounds. The inherent flexibility in marker density and the cost-effective, high-throughput nature of skim WGR provides substantial advantages, allowing more samples to be sequenced for the same cost as other traditional GBS methods, increasing the power of association studies. As a broad recommendation, a realized coverage between 1x and 2x with relatively stringent depth filtering is suggested for the application of skim WGR in canola. Crop species including soybean, safflower and tomato have genomes of a similar size to canola and so could use 1x coverage as a starting point for skim WGR under current sequencing cost structures, but this needs to be evaluated on a case-by-case basis taking into consideration sample diversity, expected heterozygosity and available resources. While skim WGR has been applied in some plant species with large genomes, for example pea, to achieve 1x coverage in this species requires 4.3 Gbp of sequencing per sample. As such there are still substantial challenges present for the implementation of skim WGR in large genomes, and ultra-low skim WGR (<1x coverage) will likely remain most suitable in biparental populations. However, as sequencing technology advances and costs continue to fall, more and more species will become amenable to the method. The presence of a reference genome, and a list of pre-validated SNPs eases the implementation of skim WGR in canola, although *de novo* SNP discovery can be performed provided a certain degree of error can be accepted. Importantly, the DHs demonstrated the difficulty of accurately genotyping canola due to homoeologous misalignment. In future, the cost-structure and accuracy of long-read sequencing may be such that it will be the preferred method for routine genotyping in highly duplicated genomes. Whole genome sequencing, whether based on short or long reads, is likely to become the GBS method that will predominate in all species due its stable, flexible and transferable nature.

## Author Contributions

DB and MM prepared the plant materials. MM, MD, MS, PT, and YO performed the sequencing library preparation. MM performed the data analysis. MM, GS, HD, and NC conceptualized the project and assisted in drafting the manuscript. All authors read and approved the final manuscript.

## Conflict of Interest Statement

The authors declare that the research was conducted in the absence of any commercial or financial relationships that could be construed as a potential conflict of interest.

## Supplementary Material

FIGURE S1 Flowchart of *in silico* skim WGR SNP set generation for the global diversity panel.

FIGURE S2 Heatmaps of SNP distribution across chromosomes for each skim level and filtering depth in the global diversity panel.

TABLE S1 Number and accuracy of SNPs for each skim level and filtering depth in the global diversity panel.
