# Supplementary material for: Evaluation and Recommendations for Routine Genotyping Using Skim Whole Genome Re-sequencing in Canola
Source: Front Plant Sci. 2018 Dec 7;9:1809. doi: 10.3389/fpls.2018.01809 (PMC6292936; doi:10.3389/fpls.2018.01809)
Supplement: FIGURE S2 — Heatmaps of SNP distribution across chromosomes for each skim level and filtering depth in the global diversity panel. [file Image_2.pdf]

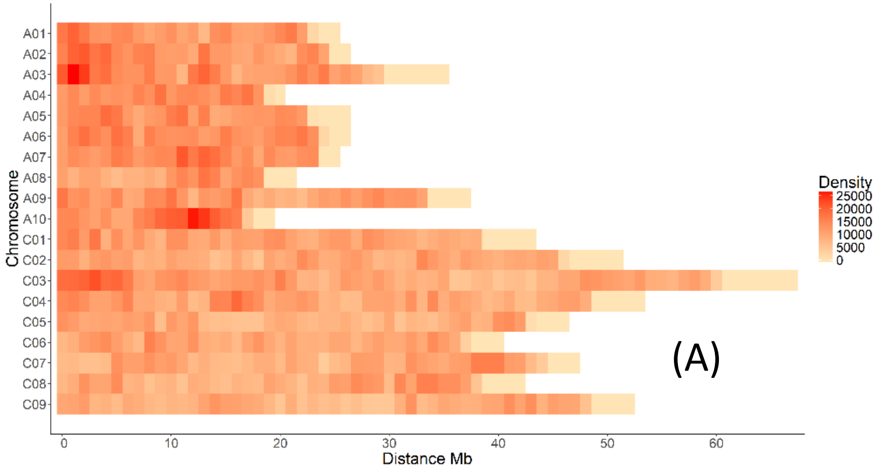

Supplementary Figure S2: Heatmaps plotting SNP density in 1 Mbp bins for each chromosome in the Darmor-*bzh* reference genome for (A) the 9 million SNP list and (B) each skim level and depth filtering combination in the SNP list called global diversity panel. Note that the scale described in the legend varies between plots.

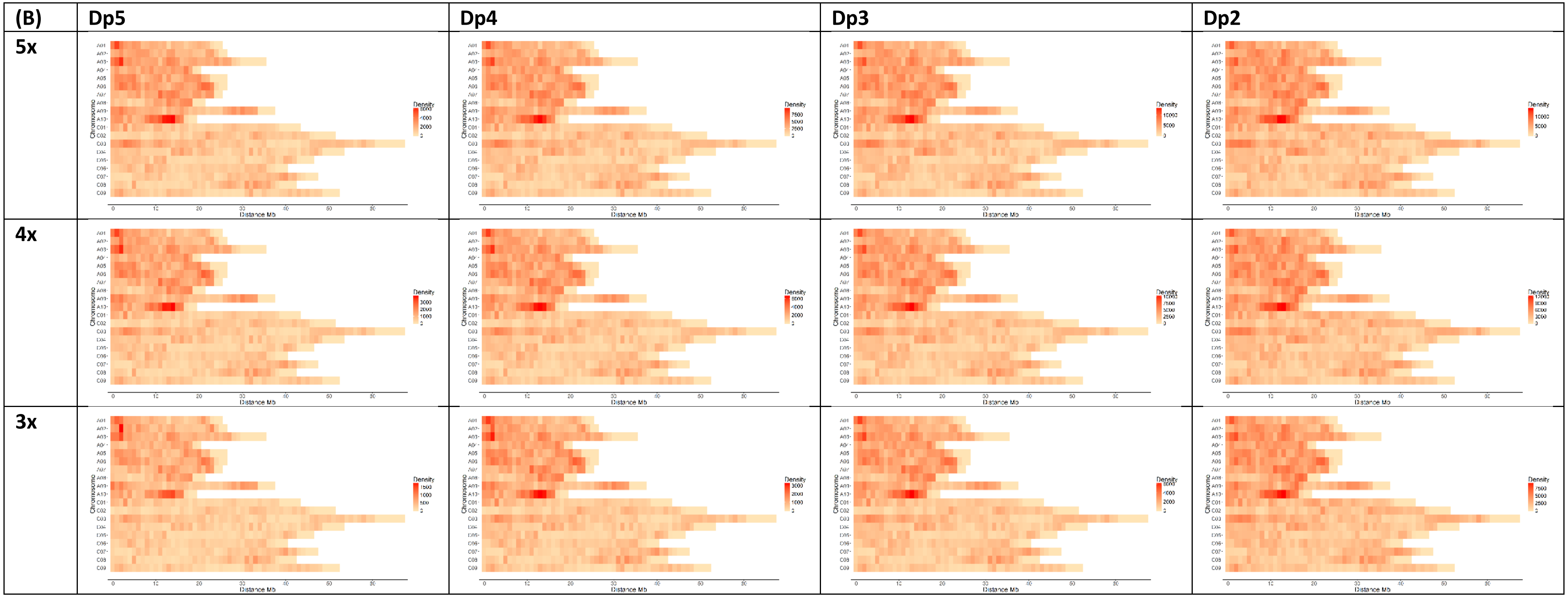

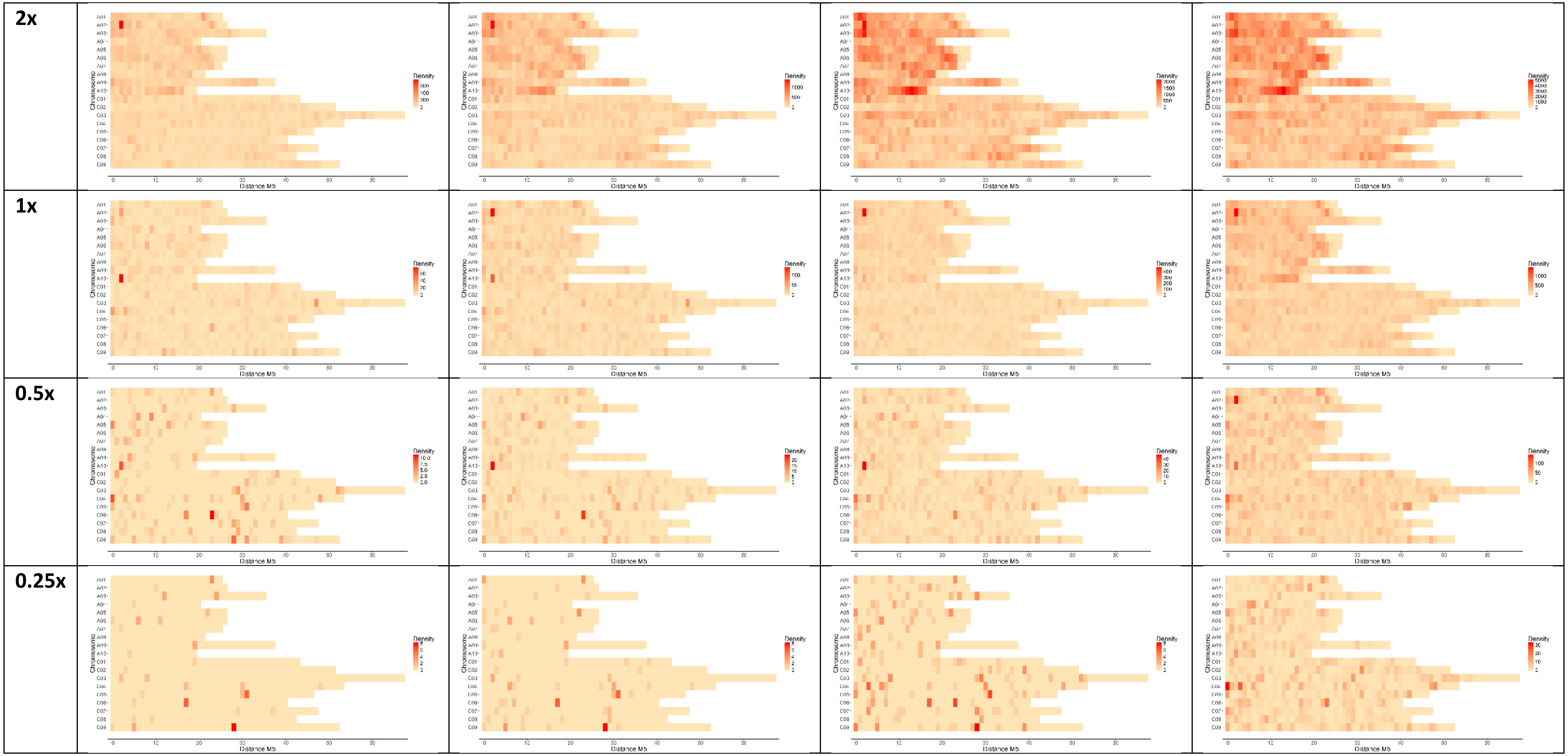
2x
1x
0.5x
0.25x
Chromosome
Distance Mb
Density
