# Supplementary material for: Evaluation and Recommendations for Routine Genotyping Using Skim Whole Genome Re-sequencing in Canola
Source: Front Plant Sci. 2018 Dec 7;9:1809. doi: 10.3389/fpls.2018.01809 (PMC6292936; doi:10.3389/fpls.2018.01809)
Supplement: TABLE S1 — Number and accuracy of SNPs for each skim level and filtering depth in the global diversity panel. [file Table_1.DOCX]

**Supplementary Table S1** Number and accuracy of SNPs retained from the approx. 9.4 million list in the global diversity panel for each skim coverage level (0.25x, 0.5x, 1x - 5x) and minimum read depth (5, 4, 3 and 2) as percentages and discrete values.

|  |  |  | **Genotype calls** | | | | | | |
| --- | --- | --- | --- | --- | --- | --- | --- | --- | --- |
| **Skim level** | **Min. read depth** | **List-based SNPs retained** | **% Missing** | **% Correct** | **% False** | **Data points** | **Missing** | **Correct** | **False** |
| 0.25x | dp 5 | 127 | 26.1 | 68.2 | 5.8 | 18,923 | 4,935 | 12,899 | 1,089 |
|  | dp 4 | 206 | 30.3 | 64.1 | 5.6 | 30,694 | 9,296 | 19,666 | 1,732 |
|  | dp 3 | 529 | 35.6 | 58.5 | 5.9 | 78,821 | 28,071 | 46,095 | 4,655 |
|  | dp 2 | 3,137 | 39.0 | 53.3 | 7.7 | 467,413 | 182,302 | 249,101 | 36,010 |
| 0.5x | dp 5 | 591 | 33.9 | 61.1 | 5.0 | 88,059 | 29,832 | 53,831 | 4,396 |
|  | dp 4 | 1,263 | 35.7 | 58.9 | 5.4 | 188,187 | 67,157 | 110,842 | 10,188 |
|  | dp 3 | 3,293 | 37.1 | 57.2 | 5.7 | 490,657 | 182,115 | 280,515 | 28,027 |
|  | dp 2 | 17,533 | 38.7 | 54.6 | 6.7 | 2,612,417 | 1,010,331 | 1,426,505 | 175,581 |
| 1x | dp 5 | 4,165 | 35.2 | 60.6 | 4.2 | 620,585 | 218,435 | 376,193 | 25,957 |
|  | dp 4 | 10,268 | 37.0 | 58.9 | 4.1 | 1,529,932 | 566,837 | 901,111 | 61,984 |
|  | dp 3 | 33,158 | 38.8 | 57.3 | 3.9 | 4,940,542 | 1,917,141 | 2,830,885 | 192,516 |
|  | dp 2 | 181,106 | 39.6 | 55.9 | 4.5 | 26,984,794 | 10,689,940 | 15,071,720 | 1,223,134 |
| 2x | dp 5 | 60,939 | 38.0 | 59.9 | 2.1 | 9,079,911 | 3,453,847 | 5,434,506 | 191,558 |
|  | dp 4 | 165,322 | 38.5 | 59.3 | 2.2 | 24,632,978 | 9,482,139 | 14,600,746 | 550,093 |
|  | dp 3 | 459,008 | 38.0 | 59.3 | 2.7 | 68,392,192 | 25,993,159 | 40,526,837 | 1,872,196 |
|  | dp 2 | 1,193,368 | 35.5 | 60.8 | 3.8 | 177,811,832 | 63,105,331 | 108,020,797 | 6,685,704 |
| 3x | dp 5 | 298,103 | 37.2 | 61.2 | 1.6 | 44,417,347 | 16,521,023 | 27,187,546 | 708,778 |
|  | dp 4 | 655,669 | 36.6 | 61.5 | 1.9 | 97,694,681 | 35,758,024 | 60,095,797 | 1,840,860 |
|  | dp 3 | 1,333,089 | 34.9 | 62.6 | 2.5 | 198,630,261 | 69,365,400 | 124,337,709 | 4,927,152 |
|  | dp 2 | 2,229,034 | 30.7 | 65.9 | 3.4 | 332,126,066 | 102,058,585 | 218,776,799 | 11,290,682 |
| 4x | dp 5 | 741,998 | 35.4 | 63.2 | 1.4 | 110,557,702 | 39,155,411 | 69,863,467 | 1,538,824 |
|  | dp 4 | 1,349,178 | 34.2 | 64.1 | 1.7 | 201,027,522 | 68,657,468 | 128,957,296 | 3,412,758 |
|  | dp 3 | 2,223,904 | 31.7 | 66.0 | 2.3 | 331,361,696 | 105,138,373 | 218,708,897 | 7,514,426 |
|  | dp 2 | 2,929,725 | 26.4 | 70.5 | 3.1 | 436,529,025 | 115,289,416 | 307,862,241 | 13,377,368 |
| 5x | dp 5 | 1,286,027 | 33.4 | 65.3 | 1.2 | 191,618,023 | 64,040,616 | 125,186,093 | 2,391,314 |
|  | dp 4 | 2,038,444 | 31.7 | 66.8 | 1.5 | 303,728,156 | 96,306,444 | 202,765,928 | 4,655,784 |
|  | dp 3 | 2,935,573 | 28.8 | 69.1 | 2.0 | 437,400,377 | 126,174,221 | 302,296,221 | 8,929,935 |
|  | dp 2 | 3,305,828 | 22.3 | 74.9 | 2.7 | 492,568,372 | 110,016,685 | 369,110,040 | 13,441,647 |
